# The relationship between the systemic immune inflammation index and the nonalcoholic fatty liver disease in American adolescents

**DOI:** 10.1186/s12876-024-03324-6

**Published:** 2024-07-23

**Authors:** Dong-fang Fu, Bin Chen

**Affiliations:** Department of Ultrasound, Hangzhou Xiaoshan First People’s Hospital, No.199, Shixin South Road, Xiaoshan District, Hangzhou, Zhejiang 311201 China

**Keywords:** Non-alcoholic fatty liver disease, Systemic immune-inflammation index, Population attributable fraction

## Abstract

**Background:**

Non-alcoholic fatty liver disease (NAFLD) is a growing health crisis in the general population of the United States (U.S.), but the relationship between systemic immune-inflammation (SII) index and NAFLD is not known.

**Methods:**

We collected data from the National Health and Nutrition Examination Survey 2017–2018. Next, propensity score matching (PSM), collinearity analysis, restricted cubic spline (RCS) plot, logistic regression, quantile regression analysis, subgroup analysis, mediation analysis, and population attributable fraction were used to explore the association of the SII with risk of NAFLD.

**Results:**

A total of 665 participants including the 532 Non-NAFLD and 133 NAFLD were enrolled for further analysis after PSM analysis. The RCS results indicated that there was a linear relationship between the SII and controlled attenuation parameter (p for nonlinear = 0.468), the relationship also existed after adjustment for covariates (p for nonlinear = 0.769). The logistic regression results indicated that a high SII level was an independent risk factor for NAFLD (OR = 3.505, 95% CI: 1.092–11.249, *P* < 0.05). The quantile regression indicated that at higher quantiles (0.90, and 0.95) the SII was significantly associated with NAFLD (*p* < 0.05). Mediation analysis indicated that alanine aminotransferase (ALT), triglycerides, and blood urea nitrogen (BUN) were partially contribute to the relationship between SII and NAFLD. The population attributable fractions indicated that 23.19% (95% CI: 8.22%, 38.17%) of NAFLD cases could be attributed to SII corresponding to 133 NAFLD cases.

**Conclusion:**

There was a positive linear relationship between the SII and the risk of NAFLD. The ALT, triglycerides, and BUN had a partial mediating effect on the relationship between the SII and NAFLD.

## Introduction

Nonalcoholic fatty liver disease (NAFLD) has become a significant public health concern due to its increasing prevalence and associated health risks. NAFLD is a spectrum of liver disorders characterized by excessive fat accumulation in the liver, which can progress to nonalcoholic steatohepatitis (NASH), fibrosis, cirrhosis, and ultimately hepatocellular carcinoma. The global prevalence of NAFLD is estimated to be around 25% [1] and among individuals with obesity, diabetes, and metabolic syndrome [2]. NAFLD also poses a serious threat to the health of adolescents. According to relevant literature, the prevalence of NAFLD among adolescents is continuously increasing. The global prevalence of NAFLD in adolescents rose from 3.73 to 4.71% in the last 20 years [3, 4]. Compared to healthy adolescents, children with this disease are more prone to cardiovascular diseases and metabolic syndrome, significantly impacting their quality of life [5, 6]. Therefore, the prevention and treatment of NAFLD in adolescents should receive adequate attention.

NAFLD is closely associated with inflammation. levels of inflammatory factors such as IL-1β, IL-6, and IL-17 are significantly increased in obese children with NAFLD [7]. The severity and composition of inflammatory infiltration are related to the steatosis and severity of NAFLD in children [8]. The Systemic Immune Inflammation (SII) index is a novel biomarker that reflects the systemic immune response and inflammation in various diseases. It is calculated based on the peripheral blood cell counts, including platelets, neutrophils, and lymphocytes, and is associated with the prognosis and outcomes of several diseases such as cancer, infectious diseases, and cardiovascular diseases [9–12]. The association of the SII with NAFLD has gained increasing attention due to the potential role of inflammation in NAFLD pathogenesis and progression [13]. Previous studies have suggested that elevated SII levels are associated with the severity of liver fibrosis in NAFLD patients [12]. Furthermore, SII has been proposed as a potential prognostic marker for NAFLD-related complications, such as hepatocellular carcinoma and cardiovascular events [13]. However, most of these studies have been conducted on adult patients with NAFLD, and the situation among adolescents is not well understood. Therefore, investigating the relationship between SII and NAFLD in adolescents may provide valuable insights into the underlying mechanisms of NAFLD progression and identify potential therapeutic targets.

In this cross-sectional study, we aim to investigate the correlation between SII and NAFLD in American adolescents, as well as its potential value in predicting NAFLD using the participant’s related information from the National Health and Nutrition Examination Survey (NHANES).

## Methods

### Data source and study participants

Data were collected from the 2017–2018 survey cycles of NHANES. It is a program of studies designed to assess the health and nutritional status of individuals in the United States and is conducted by the National Center for Health Statistics which is a part of the Centers for Disease Control and Prevention (CDC). The survey collects data through interviews, physical examinations, and laboratory tests, and is a valuable resource for studying various health and nutrition-related outcomes. 9254 participants were collected. Of these participants, we excluded 3306 without the Vibration Controlled Transient Elastography (VCTE) data. Notable, the participants aged > 12 years old were allowed to undergo VCTE. Next, the 456 participants’ elastography examination status was ineligible, not performed, or partial, which were also excluded. We further excluded 43 with serologic positivity for viral hepatitis, 592 with 4 or 5 drinks a day, 205 without SII data, and 3691 with age > 20. 961 participants including 828 Non-NAFLD and 133 NAFLD remained and were used for performed PSM analysis. Finally, 665 participants (12 years old < age < 20 years old) including 532 Non-NAFLD and 133 NAFLD were enrolled in the study.

### Diagnosis of nonalcoholic fatty liver disease

Controlled attenuation parameter (CAP) is a non-invasive technique used to assess hepatic steatosis by measuring the ultrasound attenuation in the liver. CAP has been shown to have good diagnostic accuracy for detecting hepatic steatosis in NAFLD patients [14]. The median CAP was categorized using 285 dB/m as a threshold for diagnosing liver steatosis, with optimal diagnostic performance [15].

### Calculation of systemic immune-inflammation index

The systemic immune-inflammation index was calculated based on the Lymphocyte, neutrophil, and platelet counts by the following formula: SII= (platelet count × neutrophils count)/lymphocytes count. The three kinds of cells were measured via automated hematology analyzing devices and their count was expressed as x10^3^cells/µL [16].

### Covariates

This study enrolled some potential covariates that affect the relationship between the SII and NAFLD. These covariates included age, gender, asthma, smoke, physical activity, poverty income ratio, body mass index (BMI, kg/m^2^), alanine aminotransferase (ALT, U/L), alkaline phosphatase, aspartate aminotransferase (AST, U/L), blood urea nitrogen (BUN, mg/dL), creatinine, glucose, triglycerides, uric acid, and Non-high density lipoprotein (Non-HDL) cholesterol.

### Statistical analyses

Propensity score matching (PSM) was used to eliminate bias and control for potential confounding variables. The SII was normalized by log due to the relatively large data range. The Mann-Whitney was used to compare differences in continuous characteristics between the Non-NAFLD and NAFLD groups, and the chi-square test was used to compare differences in categoric characteristics between two groups. The collinearity analysis was performed to evaluate and exclude variables with collinearity and variance inflation factor (VIF) > 5 considered to be collinear [17]. The Restricted cubic spline (RCS) curve was plotted to explore the relationship between the SII index with the NAFLD. Logistic regression analysis was used to explore the relationship between the SII with NAFLD. Quantile regression analysis was conducted to fit the correlation between SII and NAFLD better. We conducted subgroups to explore the relationship between SII and NAFLD in different subgroups via logistic regression. Stratification factors contained gender (male/female), race (Hispanic/Non-Hispanic White/Non-Hispanic Black/other races) asthma (yes/no), and physical activity (yes/no). Moreover, the interaction analysis was conducted to explore the differences between the subgroups. The mediation analysis explored the underlay mediate variable effect on the relationship between the SII and NAFLD. Population attributable fraction was conducted to quantify the NAFLD burden attributable to SII. All statistical analyses were performed using R 4.1.0, with two-tailed *P* < 0.05 indicating statistical significance.

## Results

### Participant characteristics

A total of 961 participants were included, of which 828 were Non-NAFLD, and 133 were NAFLD. There were significant differences in age, gender, physical activity, poverty income ratio, BMI, ALT, AST, triglycerides, uric acid, Non-HDL cholesterol, and Log SII between the Non-NAFLD group and NAFLD group (all *p* < 0.05). Then the PSM was performed based on 4:1, and finally, the 532 Non-NAFLD and 133 NAFLD were selected for further analysis. The results showed that there were significant differences in ALT, AST, triglycerides, Non-HDL cholesterol, body mass index, uric acid, Log SII, BUN, and poverty income ratio between the Non-NAFLD group and NAFLD group (Table 1, all *p* < 0.05). Triglycerides, uric acid, Non-HDL cholesterol, Log SII, and AST in Non-NAFLD group were significantly lower than those in the NAFLD group, while blood urea nitrogen in Non-NAFLD group was significantly higher than in the NAFLD group.

**Table 1:** Patient characteristics

	Before PSM			After PSM		
Variables	Non-NAFLD group (*n* = 828)	NAFLD group (*n* = 133)	P-value	Non-NAFLD group (*n* = 532)	NAFLD group (*n* = 133)	P-value
Age	16.00 [14.00, 18.00]	16.00 [14.00, 18.00]	0.014	16.00 [14.00, 18.00]	16.00 [14.00, 18.00]	0.828
Gender			0.003			0.810
Male	413 (49.88)	85 (63.91)		334 (62.78)	85 (63.91)	
Female	415 (50.12)	48 (36.09)		198 (37.22)	48 (36.09)	
Asthma			0.148			0.130
Yes	155 (18.72)	18 (13.53)		102 (19.17)	18 (13.53)	
No	673 (81.28)	115 (86.47)		430 (80.83)	115 (86.47)	
Smoke			0.182			0.675
Yes	21 (2.54)	3 (2.26)		19 (3.57)	3 (2.26)	
No	204 (24.64)	43 (32.33)		159 (29.89)	43 (32.33)	
Unknown	603 (72.82)	87 (65.41)		354 (66.54)	87 (65.41)	
Physical activity			0.006			0.082
Yes	716 (86.47)	103 (77.44)		86 (16.16)	30 (22.56)	
No	112 (13.53)	30 (22.56)		446 (83.84)	103 (77.44)	
Poverty income ratio	1.79 [1.00, 3.33]	1.31 [0.72, 2.72]	0.002	1.81 [1.00, 3.40]	1.31 [0.72, 2.72]	0.004
BMI (kg/m^2^)	22.10 [19.70, 26.00]	31.50 [28.30, 36.20]	< 0.001	22.10 [19.90, 25.90]	31.50 [28.30, 36.20]	< 0.001
ALT (U/L)	13.00 [10.00, 17.00]	23.00 [16.00, 39.00]	< 0.001	13.00 [11.00, 18.00]	23.00 [16.00, 39.00]	< 0.001
Alkaline phosphatase (IU/L)	104.00 [76.00, 178.00]	107.00 [80.00, 172.00]	0.699	102.00 [78.00, 159.00]	107.00 [80.00, 172.00]	0.338
AST (U/L)	18.00 [15.00, 22.00]	21.00 [18.00, 28.00]	< 0.001	18.00 [16.00, 22.00]	21.00 [18.00, 28.00]	< 0.001
BUN (mg/dL)	12.00 [9.00, 14.00]	11.00 [10.00, 14.00]	0.595	12.00 [10.00, 15.00]	11.00 [10.00, 14.00]	0.016
Creatinine (mg/dL)	0.71 [0.60, 0.83]	0.73 [0.61, 0.86]	0.266	0.76 [0.64, 0.87]	0.73 [0.61, 0.86]	0.157
Glucose (mg/dL)	88.00 [84.00, 93.00]	89.00 [85.00, 94.00]	0.052	88.00 [84.00, 93.00]	89.00 [85.00, 94.00]	0.119
Triglycerides (mg/dL)	75.00 [58.00, 101.00]	109.00 [79.00, 171.00]	< 0.001	76.00 [58.00, 105.00]	109.00 [79.00, 171.00]	< 0.001
Uric acid (mg/dL)	4.80 [4.10, 5.70]	6.00 [5.00, 7.00]	< 0.001	5.10 [4.30, 5.90]	6.00 [5.00, 7.00]	< 0.001
Non-HDL cholesterol (mg/dL)	98.00 [82.00, 117.00]	111.00 [97.00, 139.00]	< 0.001	101.00 [83.00, 118.00]	111.00 [97.00, 139.00]	< 0.001
Log SII	2.602 ± 0.230	2.682 ± 0.208	< 0.001	2.590 ± 0.227	2.682 ± 0.208	< 0.001

*Abbreviations* Non-HDL cholesterol, non-high density lipoprotein cholesterol; SII, systemic immune inflammation index. BMI, body mass index; ALT, alanine aminotransferase; AST, aspartate aminotransferase; BUN, blood urea nitrogen

**Statistical analysis method**: continuous characteristics: Mann-Whitney; categoric characteristics: the chi-square test

### SII is an independent risk factor for NAFLD

**Fig. 1:**
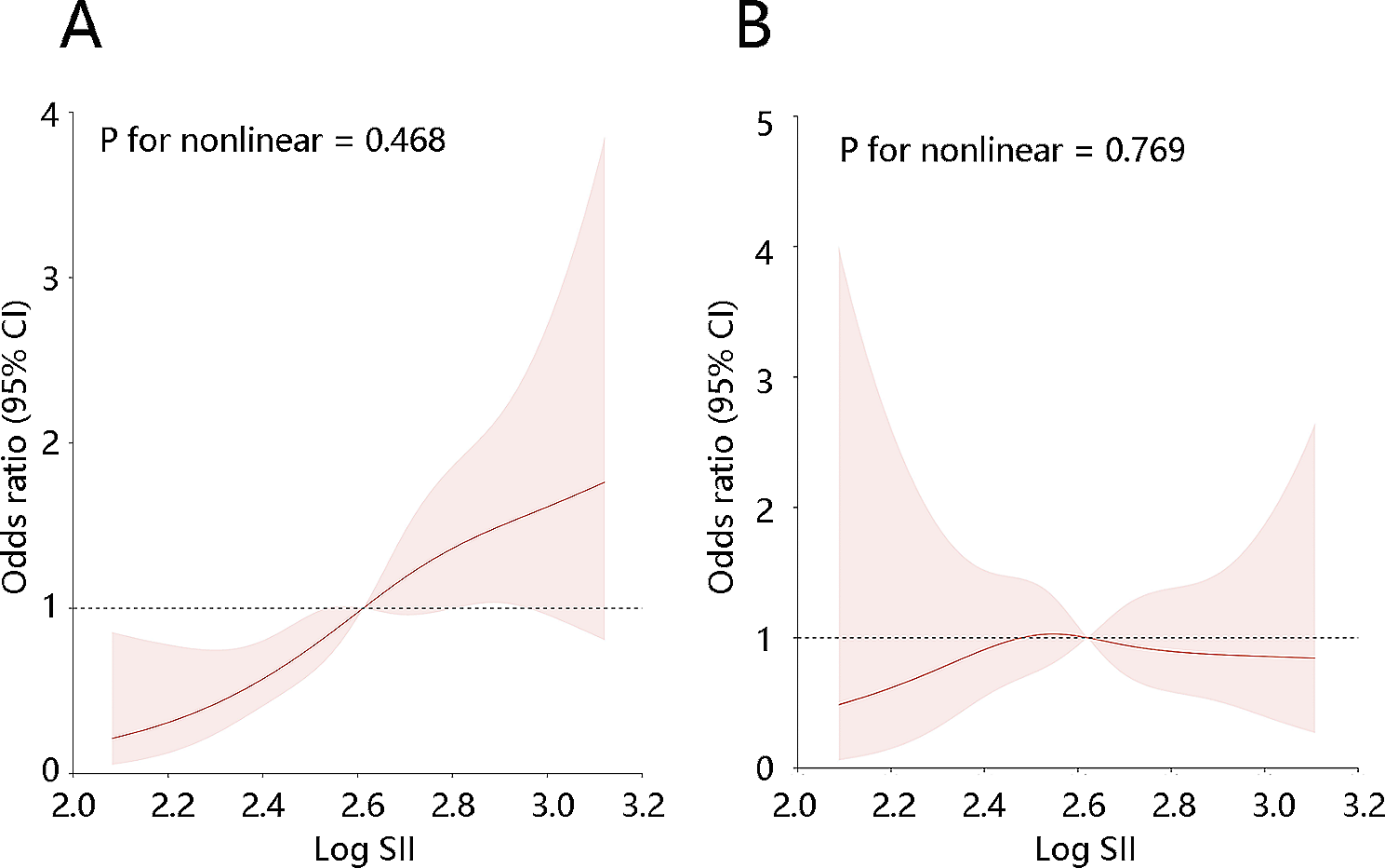
Restricted cubic spline (RCS) curve for the relationship between the SII index with the controlled attenuation parameter (CAP) in patients with NAFLD. (**A**) RCS model (**B**) RCS model after adjusting for ALT, triglycerides, Non-HDL cholesterol, body mass index, uric acid, BUN, and poverty income ratio

To acquire the precise relationship between the SII and NAFLD, the collinearity analysis was performed before the RCS analysis. The collinearity analysis results showed no collinear relationships among the variables which had differences with statistical significance between the Non-NAFLD and NAFLD and obtained from Table 2(Table 2, all VIF < 5. Further, the RCS results indicated that there was a linear relationship between the SII and CAP (Fig. 1A, P for nonlinear = 0.468), the relationship also existed after adjustment for ALT, AST, triglycerides, Non-HDL cholesterol, uric acid, BUN, and poverty income ratio (Fig. 1B, P for nonlinear = 0.769). Therefore, the logistic regression was performed. The results of the univariate logistic regression indicated that high levels of SII were a risk factor for NAFLD (OR = 6.415, 95% CI: 2.655–15.504, *P* < 0.001, Table 3). After adjustment for significant factors identified in the baseline characteristics including ALT, AST, triglycerides, Non-HDL cholesterol, uric acid, BUN, and poverty income ratio, high SII level was an independent risk factor for NAFLD (OR = 3.505, 95% CI: 1.092–11.249, *P* < 0.05, Table 3).

**Table 2:** Collinearity analysis

Variables	VIF
ALT	2.103
AST	1.729
Triglycerides	1.568
Non-HDL cholesterol	1.545
BMI	1.400
Uric acid	1.263
Log SII	1.116
BUN	1.061
Poverty income ratio	1.030

*Abbreviations* SII, systemic immune inflammation index; Non-HDL cholesterol, non-high density lipoprotein cholesterol; VIF, variance inflation factor; BMI, body mass index; ALT, alanine aminotransferase; AST, aspartate aminotransferase; BUN, blood urea nitrogen

**Statistical analysis method**: collinearity analysis

**Table 3:** Logistic regression analysis of the association of SII with nonalcoholic fatty liver disease

	Univariate analysis		Multivariate analysis	
Variables	OR (95% CI)	P-value	OR (95% CI)	P-value
ALT	1.066 (1.049–1.083)	< 0.001	1.066 (1.040–1.093)	< 0.001
AST	1.023 (1.006–1.039)	0.007	0.971 (0.940–1.004)	0.082
Triglycerides	1.010 (1.007–1.013)	< 0.001	1.009 (1.004–1.014)	< 0.001
Uric acid	1.552 (1.338–1.801)	< 0.001	1.129 (0.937–1.361)	0.201
Non-HDL cholesterol	1.017 (1.010–1.024)	< 0.001	0.994 (0.984–1.004)	0.241
BUN	0.920 (0.866–0.977)	0.007	0.908 (0.839–0.983)	0.017
Poverty income ratio	0.821 (0.712–0.947)	0.007	0.850 (0.721–1.001)	0.051
Log SII	6.415 (2.655–15.504)	< 0.001	3.505 (1.092–11.249)	0.035

*Abbreviations* SII, systemic immune inflammation index; Non-HDL cholesterol, non-high density lipoprotein cholesterol; BMI, body mass index; ALT, alanine aminotransferase; AST, aspartate aminotransferase; BUN, blood urea nitrogen; OR (95% CI), odds ratio (95% confidence interval)

For further exploring the relationship between the SII and NAFLD, the quantile regression was fitted on the 0.05,0.10,0.25,0.75,0.9,0.95 of SII distribution to investigate the relationship between the CAP and SII. The results indicated that at higher quantiles (0.90, and 0.95) the SII was significantly associated with NAFLD (Table 4*p* < 0.05).

**Table 4:** Quantile regression analysis of the association of SII with nonalcoholic fatty liver disease

Quantile	Crude coefficients (95% CI)	*P*-value	Adjusted coefficients (95% CI)	*P*-value
0.05	5.432 (-29.350, 28.776)	0.775	-3.119 (-30.102-45.480)	0.884
0.10	11.053 (-6.017, 33.689)	0.312	8.551 (-22.615-29.533)	0.423
0.25	19.348 (7.925, 32.456)	0.033	15.443 (-2.582-25.122)	0.078
0.50	24.633 (5.653, 41.686)	0.006	16.966 (2.962–35.175)	0.042
0.75	50.754 (38.194, 71.734)	< 0.001	19.287 (12.306–42.303)	0.070
0.90	77.224 (50.567, 110.504)	< 0.001	40.325 (14.272–71.487)	0.005
0.95	76.683 (42.406, 130.475)	0.001	41.039 (6.414–67.081)	0.016

*Abbreviations* SII, systemic immune inflammation index; 95% CI, 95% confidence interval

### Subgroup analysis

To further the relation between the SII and NAFLD, a subgroup analysis was performed, which was stratified by gender, race, asthma, and physical activity. We selected those four factors for further subgroup analyses because they were less studied, but important for NAFLD based on reported literatures [18–20]. The results indicated that SII was a risk factor for NAFLD events in male/female NAFLD patients, Non-Hispanic Black patients, patients with/without asthma, and patients without physical activity (*p* < 0.05, Table 5). Moreover, the interaction analysis showed that there was no statistical significance for gender, race, asthma, and physical activity (all P for interactions > 0.05, Table 5).

**Table 5:** Association of SII with nonalcoholic fatty liver disease in different subgroups

Variables	OR (95% CI)	*P*-value	*P* for interaction
Gender			0.903
Male	7.276 (2.364, 22.389)	0.001	
Female	7.878 (1.636, 37.935)	0.010	
Race			0.590
Hispanic	3.249 (0.798, 13.218)	0.100	
Non-Hispanic White	6.530 (0.980, 43.509)	0.052	
Non-Hispanic Black	9.504 (1.191, 75.840)	0.798	
other races	7.681 (0.796, 75.840)	0.028	
Asthma			0.693
Yes	9.946 (1.205, 82.093)	0.033	
No	5.844 (2.198, 15.535)	< 0.001	
Physical activity			0.711
Yes	1.258 (0.216, 7.318)	0.799	
No	10.395 (3.718, 29.065)	< 0.001	

*Abbreviations* SII, systemic immune inflammation index; OR (95% CI), odds ratio (95% confidence interval)

**Statistical analysis method**: logistic regression analysis

### Mediation analyses and population attributable fraction

**Fig. 2:**
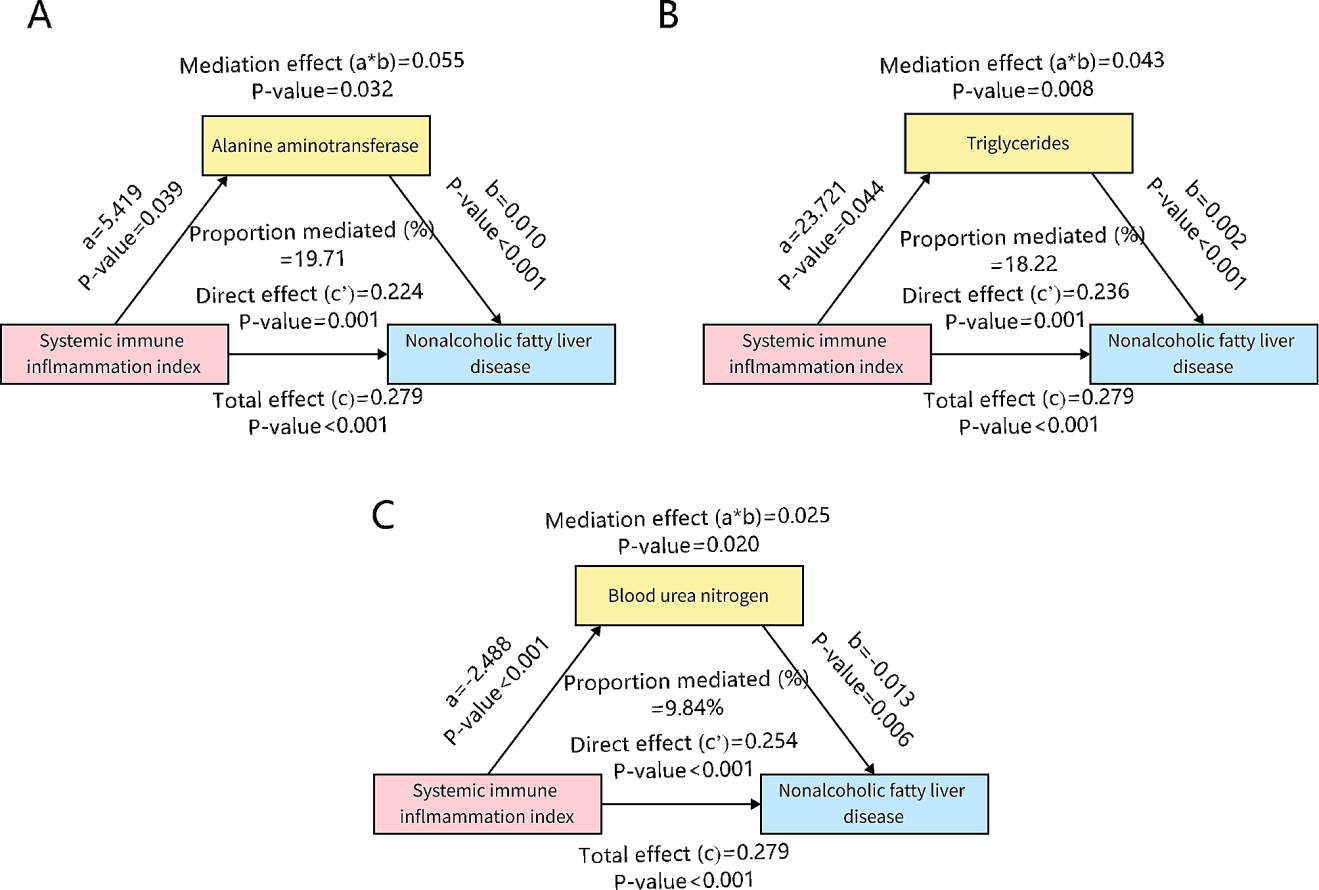
The association of the SII with mediation variables and NAFLD. (**A**) ALT was the mediation variable. (**B**) The Triglycerides was the mediation variable. (**C**) The BUN was the mediation variable

The multivariable logistic regression results showed that ALT, triglycerides, and BUN were independent risk factors for NAFLD. Mediation analysis was performed to further explore their function in the NAFLD. The results indicated that three variables partially contributed to the relationship between SII and NAFLD. ALT, triglycerides, and BUN mediated 19.71%,18.22%, and 9.84% of the effect of SII on NAFLD (Fig. 2A-C). Attributable cases (AT) and population attributable fractions (PAF) showed NAFLD burden attributable to SII (Table 6). 23.19% (95% CI: 8.22%, 38.17%) of NAFLD cases can be attribute to SII corresponding to 133 NAFLD cases.

**Table 6:** Estimated burden of NAFLD attributable to SII among adolescents

	No. of NAFLD cases	Attributed cases (95% CI)	Population attributable fraction, % (95% CI)
NHANES	133	31 [11, 51]	23.19 (8.22, 38.17)

## Discussion

In this study, we found that there was a positive linear relationship between the SII and NAFLD in American adolescents. In addition, ALT, triglycerides, and BUN had a partial mediating effect on the relationship between the SII and NAFLD.

The pathogenesis of NAFLD is a complex process involving the interaction of multiple factors such as genetics, environment, and metabolic abnormalities. Most related studies have shown that the mechanisms are different and include [1] Disorders of fat metabolism and Inflammatory response: The main feature of NAFLD is the accumulation of lipids in the liver, mainly triacylglycerol. This may be due to metabolic abnormalities in the synthesis and transport of fats in the liver. Fat accumulation triggers an inflammatory response in the liver. Activation of inflammatory cells and cytokines leads to inflammatory responses in liver tissue, including hepatocyte injury and apoptosis [21, 22]. [2] Oxidative stress and mitochondrial dysfunction: Oxidative stress and mitochondrial dysfunction also play an important role in the development of NAFLD. Cellular damage caused by lipid peroxidation and oxidative stress may increase the risk of NAFLD progression [23, 24]. [3] Liver fibrosis and scarring: Fibrosis is the excessive accumulation of collagen in liver tissue, and scarring is when fibrous tissue replaces normal liver tissue. [4] Interaction of genes with environmental factors: Genetic and environmental factors are also thought to have an impact on the development and progression of NAFLD. Some genetic mutations may increase the risk of developing NAFLD, while environmental factors such as diet, lifestyle, obesity, and metabolic syndrome increase the risk of developing NAFLD [25].

The “multiple-hit model” is currently the widely accepted theory for NAFLD in adolescents [26]. This model suggests that NAFLD results from the interplay of genetic and environmental dietary factors, along with crosstalk between various organs and tissues, leading to extensive metabolic dysfunction. The initial step in NAFLD for both adults and adolescents involves triglyceride accumulation and insulin resistance (IR) [27–30]. Our study indicates that triglyceride levels are significantly higher in NAFLD patients compared to those without the condition. Triglycerides are formed by the esterification of glycerol and free fatty acids (FFAs). The accumulation of triglycerides suggests an excess of FFAs in the liver, which, when activated by acetyl-CoA synthase, may trigger esterification or β-oxidation pathways. This can lead to oxidative stress, generating reactive oxygen species and resulting in mitochondrial dysfunction [31, 32]. This dysfunction activates inflammatory pathways such as JNK-AP-1 and IKK-NF-κBD [33]. The production of inflammatory cytokines can induce IR. Hepatic IR prevents insulin from inhibiting lipolysis, leading to reduced fatty acids [34], lower hepatic acetyl-CoA levels, and reduced pyruvate carboxylase activity, thereby inhibiting the conversion of pyruvate to glucose [35]. These processes activate the production of pro-inflammatory cytokines in the liver, exposing it to high levels of these cytokines and leading to histological changes similar to non-alcoholic steatohepatitis [36].

SII is reminiscent of the interaction of thrombocytosis, inflammation, and immunity. Song et al. indicated that SII could be used as a simple and affordable way to identify hepatic steatosis because there was a positive relationship between SII and hepatic steatosis [37]. The pathological progression of NAFLD follows three steps including steatosis, lipotoxicity, and inflammation [38]. In our study, we found that the patients with high SII had a high risk of NAFLD. SII, secreted by lymphocytes and central granulocytes, leads to NAFLD through the release of inflammatory factors such as Tumor Necrosis Factor-alpha (TNF-alpha), Interferon-gamma (IFN-gamma), Interleukin 2(IL-2), Interleukin 2 (IL-6), Interleukin 1 (IL-1), etc. [39–41]. These inflammatory factors can trigger the generation of oxidative stress, disrupting the cellular redox balance and producing excessive reactive oxygen species, such as free radicals. Excessive oxidative stress can damage cell membranes, proteins, and nucleic acids, leading to cell injury and death. This cell damage, in turn, can provoke more inflammatory reactions, forming a vicious cycle and causing changes in liver cell function [42–44]. In our study, there was a linear relationship between the SII and the NAFLD. The result was not consistent with the Zhao et al. Their research showed that the relationship between the SII and the risk of NAFLD was U-shaped [45]. There was a difference in the definition of NAFLD. NAFLD was defined using the US fatty liver index (FLI) in their study, while we used the CAP to diagnose the NAFLD. In addition, our study population was adolescents, while their study population was adults. In the study of Xie et al., the CAP was used to diagnose hepatic steatosis and they found that there was an inverted U-shaped relationship between SII and CAP [46]. This difference is likely due to dietary differences in adolescentss and adults, particularly sugar intake. Pediatric/adolescents consume more sugar compared to adults. Studies have shown that excessive sugar intake can lead to increased subclinical inflammation and is positively correlated with certain inflammatory markers [47, 48]. For example, fructose-induced liver fat accumulation involves stress pathways, leading to increased gluconeogenesis, fat synthesis, and reduced fat oxidation [49–51]. Additionally, long-term fructose exposure in the gut can trigger inflammation by increasing serum TNF-α levels [52], thereby affecting the gut-liver axis and influencing the development of NAFLD [26, 53].

We also found that ALT, triglycerides, and BUN had a partial mediating effect on the relationship between the SII and NAFLD. ALT plays a crucial role in amino acid metabolism, particularly in the reciprocal conversion between alanine and alpha-ketoglutarate. ALT is capable of transferring the amino group from alanine to alpha-ketoglutarate, generating glutamate and pyruvate. This reaction plays a key role in protein metabolism and energy metabolism. SII responds to an index of inflammation and immune balance in the body. When the SII is high, it triggers oxidative stress and produces excess ROS, which in turn damages liver cells, resulting in an increase in ALT, which means that the liver is inflamed. Inflammatory factors can lead to IR, indicating a reduced response of liver cells to insulin. Insulin typically inhibits the activity of ALT. However, in IR states, this inhibitory effect is diminished, leading to an elevation in ALT levels. Additionally, when liver cells are damaged or undergo cell death, ALT is released into the bloodstream [33, 54–57]. A higher SII means that the patient has a high level of inflammation in the body and more inflammatory factors. Some inflammatory factors, such as IL-6 and TNF-alpha, can promote an increase in lipid synthesis within liver cells, leading to the accumulation of triglycerides in liver cells. Additionally, inflammatory factors can interfere with the insulin signaling pathway, causing IR. Insulin resistance is an important hormone that inhibits lipid breakdown and clearance, resulting in its accumulation within liver cells [33, 54, 58]. These processes contribute to the occurrence and development of NAFLD. An elevation in triglyceride levels may impact the filtration function of the kidneys. In situations where triglyceride levels are elevated, the kidneys may need to process more metabolic products, including urea nitrogen. Therefore, elevated triglycerides may be associated with an increase in urea nitrogen levels. Insulin resistance and abnormalities in glucose metabolism contribute to these metabolic changes, potentially affecting the production and clearance of urea nitrogen [59, 60]. An increase in urea nitrogen levels may be a characteristic feature of NAFLD. In summary, SII may mediate inflammatory pathways and affect the levels of the three, thereby exacerbating inflammation or insulin resistance and promoting disease progression. However, the specific detailed mechanism needs to be further explored by experiments.

Although the study used a variety of data analysis methods to obtain relatively accurate conclusions in American adolescents, there were still some limitations. It should be noted that the NHANES dataset is based on cross-sectional data, which limits the ability to establish causality. Additionally, the data is self-reported and subject to recall and reporting biases, which may introduce measurement errors in the analysis. In addition, only one cycle of study data was enrolled because of year limitations, which may improve the bias of data. Lastly, the use of CAP instead of biopsy to diagnose NAFLD in adolescents is controversial and may affect the accuracy of the conclusions. These limitations should be considered when interpreting the results of this study.

In summary, there was a positive linear relationship between the SII and the risk of NAFLD. The ALT, triglycerides, and BUN had a partial mediating effect on the relationship between the SII and NAFLD. The potential mechanism of SII affecting NAFLD events needs further experimental exploration.

## Data Availability

The datasets generated during and/or analyzed during the current study are available from the corresponding author on reasonable request.
